# Supplementary material for: A yeast phenomic model for the influence of Warburg metabolism on genetic buffering of doxorubicin
Source: Cancer Metab. 2019 Oct 23;7:9. doi: 10.1186/s40170-019-0201-3 (PMC6806529; doi:10.1186/s40170-019-0201-3)
Supplement: Supplementary file 9 — Additional file 9. GO term-specific heatmaps for REMc/GTF-enriched clusters. GO term-specific heatmaps for significant GO process terms were generated as described in methods and Figs. 3 and 4. Any related child terms are presented in subsequent pages of the parent file name. GO terms with more than 100 children, with 2 or fewer genes annotated to the term, or a file size over 300KB are not shown. All heatmaps are generated with the same layout (see Figs. 3 and 4). [file 40170_2019_201_MOESM9_ESM.bz2 › Additional_File9_GOTermHeatmaps/Additional_File9_GOTermHeatmap/regulation_of_meiotic_DNA_double-strand_break_formation.pdf]

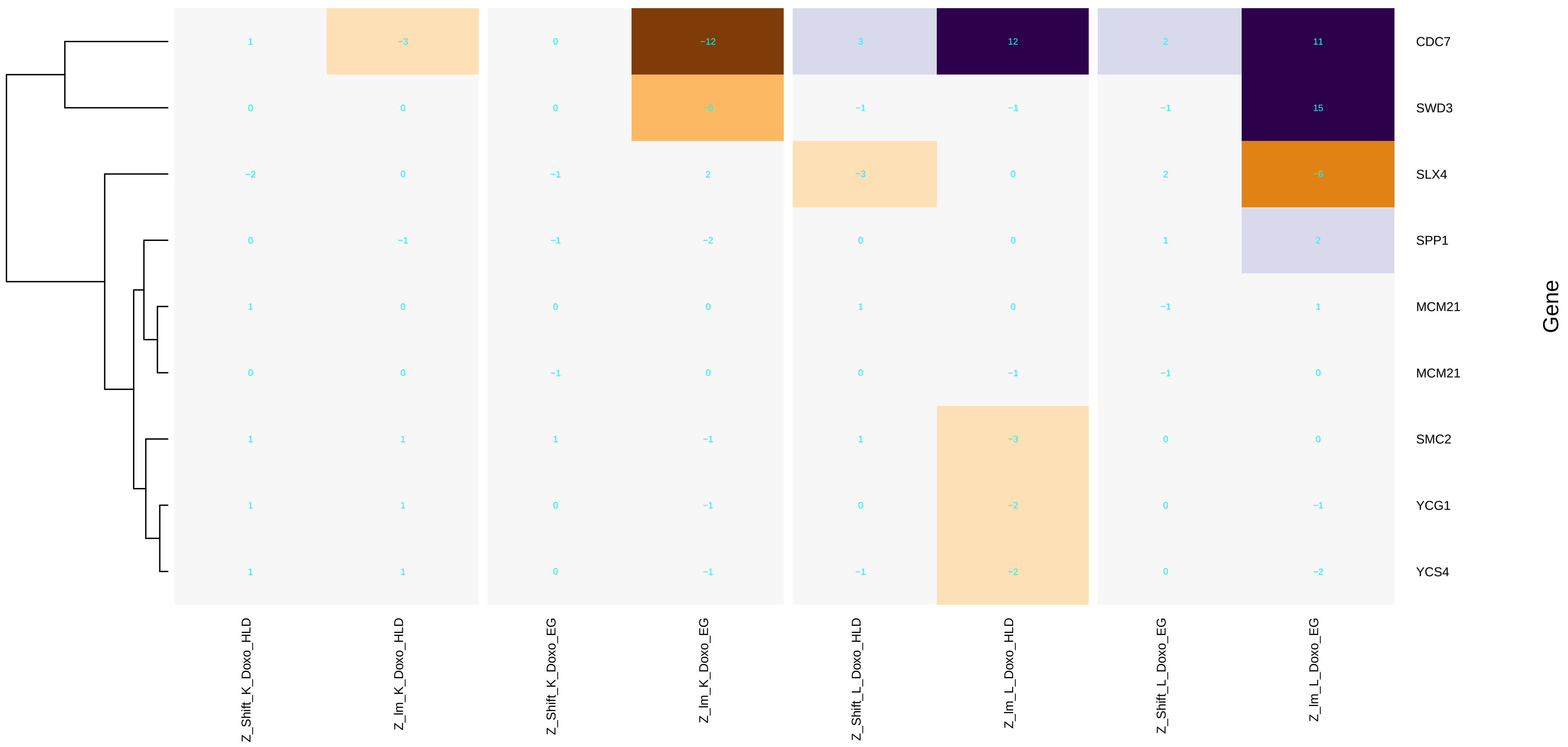

CDC7
SWD3
SLX4
SPP1
MCM21
MCM21
SMC2
YCG1
YCS4
Gene
Z_Shift_K_Doxo_HLD
Z_Im_K_Doxo_HLD
Z_Shift_K_Doxo_EG
Z_Im_K_Doxo_EG
Z_Shift_L_Doxo_HLD
Z_Im_L_Doxo_HLD
Z_Shift_L_Doxo_EG
Z_Im_L_Doxo_EG
1 −3 0 −12 3 12 2 11
0 0 0 −6 −1 −1 −1 15
−2 0 −1 2 −3 0 2 −6
0 −1 −1 −2 0 0 1 2
1 0 0 0 1 0 −1 1
0 0 −1 0 0 −1 −1 0
1 1 1 −1 1 −3 0 0
1 1 0 −1 0 −2 0 −1
1 1 0 −1 −1 −2 0 −2
